# Assessment of the performance of the plasma separation card for HIV-1 viral load monitoring in South Africa

**DOI:** 10.1128/jcm.01649-23

**Published:** 2024-03-12

**Authors:** Lucy Chimoyi, Lucia Hans, Matthew Oladimeji, Gurpreet Kindra, Karidia Diallo, Kassahun Ayalew, Geoffrey K. Setswe, Sergio Carmona

**Affiliations:** 1 Implementation Research Division, The Aurum Institute, Johannesburg, South Africa; 2 Department of Molecular Medicine and Hematology, National Health Laboratory Service, Johannesburg, South Africa; 3 Department of Molecular Medicine and Hematology, Faculty of Health Sciences, University of the Witwatersrand, Johannesburg, South Africa; 4 Division of Global HIV and Tuberculosis, Center for Global Health, Centers for Disease Control and Prevention, Pretoria, South Africa; 5 Department of Health Studies, University of South Africa, Pretoria, South Africa; 6 Foundation for Innovative New Diagnostics (FIND), Geneva, Switzerland; St Jude Children's Research Hospital, Memphis, Tennessee, USA

**Keywords:** Plasma separation card, clinical evaluation, HIV viral load monitoring, South Africa

## Abstract

Scaling up of newer innovations that address the limitations of the dried blood spot and the logistics of plasma monitoring is needed. We employed a multi-site, cross-sectional assessment of the plasma separation card (PSC) on blood specimens collected from all consenting adults, assenting young and pediatric patients living with HIV from 10 primary healthcare clinics in South Africa. Venous blood for EDTA-plasma samples was collected and analyzed according to the standard of care assay, while collected capillary blood for the PSC samples was analyzed using the Roche COBAS AmpliPrep/Cobas TaqMan (CAP/CTM) HIV-1 Test at the National Reference laboratories. McNemar tests assessed the differences in concordance between the centrifuged plasma and dried plasma spots. The usability of PSC by blood spotting, PSC preparation, and pre-analytical work was assessed by collecting seven-point Likert-scale data from healthcare and laboratory workers. We enrolled 538 patients, mostly adults [*n* = 515, 95.7% (95% CI: 93.7%–97.1%)] and females [*n* = 322, 64.2% (95% CI: 60.0%–68.1%)]. Overall, 536 paired samples were collected using both PSC- and EDTA-plasma diagnostics, and 502 paired PSC- and EDTA-plasma samples assessed. Concordance between the paired samples was obtained for 446 samples. Analysis of these 446 paired samples at 1,000 copies per milliliter threshold yielded an overall sensitivity of 87.5% [95% CI: 73.2%–95.8%] and specificity of 99.3% [95% CI: 97.9%–99.8%]. Laboratory staff reported technical difficulties in most tasks. The usability of the PSC by healthcare workers was favorable. For policymakers to consider PSC scale-up for viral load monitoring, technical challenges around using PSC at the clinic and laboratory level need to be addressed.

**IMPORTANCE:**

Findings from this manuscript emphasize the reliability of the plasma separation card (PSC), a novel diagnostic method that can be implemented in healthcare facilities in resource-constrained settings. The agreement of the PSC with the standard of care EDTA plasma for viral load monitoring is high. Since the findings showed that these tests were highly specific, we recommend a scale-up of PSC in South Africa for diagnosis of treatment failure.

## INTRODUCTION

With the global scale-up of antiretroviral therapy (ART), providers to people living with HIV (PLHIV)-related illnesses in resource-limited settings face challenges in monitoring patients on ART for treatment failure (1, 2). The World Health Organization (WHO) recommends HIV viral load (VL) monitoring at 6 months and 12 months after ART initiation and annually thereafter (3). While VL monitoring is the standard of care in low- and middle-income countries, its availability remains limited in many settings, often constrained by cost, technologies, or human resources. In South Africa, challenges with VL monitoring associated with the collection, storage, and transportation of plasma samples have been reported (4). Specimen transport requirements are known to be a significant barrier to the widespread use of VL monitoring since whole blood or centrifuged plasma samples are required to be transported to the laboratory using a stringent cold chain. To address these challenges, several strategies, including the development of point-of-care devices that have allowed for rapid, on-site VL measurements, have been implemented, but these still require transportation logistics and laboratory capacities (4–6).

Innovations in HIV VL monitoring that use dried blood spot (DBS) techniques rather than plasma have been evaluated and have shown increased access to VL testing in resource-constrained settings (7). DBS can be stored at room temperature for several days prior to transport and can be transported to the testing laboratory in an envelope via post or courier without a cold chain. However, some have raised concerns about VL testing using DBS. This testing may be prone to misclassifying patients as having failed HIV treatment who may then erroneously be required to switch ART regimens (8). It is thought that the volume of whole blood collected for the spots may be inadequate, decreasing the sensitivity of assays and producing false negatives. Furthermore, there is a possibility of overestimation of VL due to the amplification of proviral DNA (9, 10). To overcome this limitation, McClernon and McClernon (11) evaluated the performance of a novel prototype composed of a primary separation membrane and secondary absorbent wick with similar features as the DBS but can be used with downstream DNA (7) and RNA molecular-based assays (11). A recent study by Carmona et al. (7) examined the performance of this novel plasma collection device, the COBAS Plasma Separation Card (PSC; Roche Molecular Diagnostics, Pleasanton, CA, USA), which produces plasma spots rather than whole blood using mostly laboratory prepared PSCs (7). Using paired EDTA-plasma samples, the card’s bias and misclassification at a VL threshold of 1,000 copies per milliliter (cp/mL), as well as its usability, limits of detection (LoD), and stability, were examined. The study reported that PSC retained the sample collection and transport advantages of the DBS but had improved sensitivity, specificity, and reliability (7).

Although VL from PSC samples prepared in reference laboratories has shown to yield similar results as those from EDTA-plasma samples, the use of PSC collected under field conditions has not been evaluated yet in South Africa. Our study was aimed at evaluating the use of PSC (from capillary blood) collected under field conditions during routine patient care for targeted VL monitoring among adults and children on ART for at least 6 months.

## MATERIALS AND METHODS

### Study design and setting

We conducted a cross-sectional study from July to September 2019 in 10 public healthcare facilities in Gauteng (urban) and North West (rural) Provinces, South Africa. We purposely selected primary healthcare facilities with a monthly head count >4,000 patients to allow for diversity in age, setting (i.e., rural/urban), biological sex, and collection of high volumes of VL samples (>30 samples) a day.

### Study participants

The population consisted of male and female HIV-positive clients aged 2 years and older, who were currently receiving ART from the primary healthcare facilities in the two health districts. Simple random sampling techniques were applied, with proportional allocation of samples for each location, to meet the required sample sizes. A sampling frame was developed using a complete line listing of HIV clients for each location. In cases where developing a line listing of patients living with HIV for each location was difficult to obtain, convenience sampling was adopted ensuring minimum samples of adults [males (40%) and females (60%)], and children were achieved across primary healthcare facilities

### Sample size

The sample sizes were determined using an SD(s) of VL between 0.1 and 0.3 log_10_, lower and upper limits of 0.02, mean difference of 0, power of 80%, and alpha level of 5%. For a sample size of 270, adjusting the non-response rate at 20% and accounting for the design effect of 2.0 gave a recommended sample size of 540. The targeted enrollment number was a total of 780 comprising 660 adults and 120 children. Adult and child participants were to be enrolled sequentially at the selected sites until the desired sample size was reached.

### Laboratory testing

PSC testing was performed at the National Health Laboratory Service (NHLS) HIV Molecular Laboratory, Charlotte Maxeke Johannesburg Academic Hospital in Johannesburg in South Africa using the COBAS AmpliPrep/Cobas TaqMan (CAP/CTM; Roche Molecular Diagnostics, Pleasanton, CA, USA).

Routine EDTA-plasma testing was performed at the NHLS HIV VL laboratory to which the healthcare facility routinely referred VL samples. In North West province, these samples were sent to the NHLS Tshepong Hospital Laboratory, Klerksdorp in Rustenburg, whereas those from Ekurhuleni were sent to the NHLS laboratory at Charlotte Maxeke Johannesburg Academic Hospital or Dr George Mukhari Hospital, Pretoria. Plasma VL tests were performed using the CAP/CTM, the cobas HIV-1 Quantitative nucleic acid test for use on the COBAS 6800/8800 Systems (c8800; Roche Molecular Diagnostics, Pleasanton, CA, USA), or Abbott RealTime HIV-1 (m2000; Abbott Molecular Inc, Illinois, USA) assays, dependent on the assay in use at the laboratory.

### Data collection procedures

#### Plasma separation card

We followed data collection procedures as previously described in the study by Carmona et al. (8).

#### Sample collection and preparation

Healthcare workers (HCWs) in primary healthcare facilities collected approximately 2 (pediatric) to 4 (adult) mL of venous whole blood samples in EDTA tubes by venipuncture. A routine sample of capillary blood was collected from the finger using a safety lancet (Greiner bio-one 120 MiniCollect Safety Lancet, with a penetration depth of 2.0 mm) from patients attending routine HIV care. The nurse/phlebotomist collected blood using an EDTA-coated capillary tube positioned directly beneath the puncture site. A total of ~420 µL total was collected for each patient. Each PSC contains three (140 µL) wells where the blood was spotted on from the capillary tube. Caution during blood spotting by nurses/phlebotomists was made to prevent disruption or tearing of the plasma membrane. PSCs were placed in a single-usage drying rack for a minimum of 4 hours at room temperature at the collection site. Upon drying, they were packed in separate gas impermeable zip-lock bags with a desiccant and transported to the laboratory at room temperature every 2 days.

Once at the laboratory, the PSCs were stored at room temperature till use, ranging from 6 to 8 weeks after sample collection PSCs were processed according to the manufacturer’s instructions. Briefly, in a laminar flow hood, the top membrane (spotting layer) was manually removed by pulling on a removable flap on the PSC and discarded. A single spot was removed with sterile tweezers and transferred into an S-Input tube, to which 1,100 µL of CAP/CTM Specimen Pre-Extraction (SPEX) Reagent was added. Tubes were placed in a pre-heated Eppendorf thermomixer (Hamburg, Germany) and incubated for 10 minutes at 56°C and at 1,000 rpm. Thereafter, the tubes were capped and loaded onto the CAP/CTM for testing.

EDTA-plasma samples were analyzed in single determinations using the referral laboratory multiple VL systems. Handling of systems, specimens, controls, and reagents for the plasma VL was carried out according to procedures described in the respective instructions for use and the relevant NHLS assay-specific standard operating procedures which recommend storage in the primary EDTA tube at 2°C–25°C, centrifugation at 800–1,600 g for 20 minutes and transfer of plasma into a sterile polypropylene tube within 24 hours from collection for all assays.

### PSC usability assessment

HCWs who collected PSC samples at the primary healthcare clinics and laboratory staff who assessed the preparation of the PSC before testing completed a seven-point Likert-type questionnaire covering usability statements for PSC and its associated workflows. Participants scored “0” strongly disagree, “1” disagree, “2” somewhat disagree, “3” neutral, “4” somewhat agree, “5” agree, and “6” “strongly agree to the statement. This questionnaire was completed once after sample collection was completed for all consenting patients. For the usability assessment, HCWs evaluated the collected blood samples and spotted the PSC, while the laboratory staff assessed the processing of the PSC for VL after receiving them.

### Data analysis

All study data were collected on hand-held mobile devices (Lenovo TB X304L Android version 8.1.0). Statistical analysis was performed using Stata software, Version 15 (StataCorp, College Station, TX, USA). Statistical power was set at 80% and alpha at 5%, and all tests of hypotheses were two-directional, where applicable. We calculated the sensitivity and specificity of PSC with EDTA samples using the WHO-recommended threshold for treatment failure (≥1,000 cp/mL). Also, we used the paired *t* test to compare VL-detectable numerical results between the PSC and EDTA. In addition, we conducted a McNemar test to compare the rate of agreement between both tests by evaluating if there is a statistical difference in the marginal frequencies of their viral suppression (VL of <1,000 cp/mL) results. Finally, we computed the confidence intervals for the respective values using their proportions. Likert-scale responses were summarized, and the mean score per statement was computed.

## RESULTS

### Study participants’ characteristics

A total of 538 patients were presented at the clinics for routine visits and were eligible for HIV VL monitoring. Of these, demographic and clinical characteristics by age group were summarized for 502 participants whose PSC and EDTA samples were paired (Table 1). The participants were mostly female (*n* = 322, 64.2%) and had been on treatment for more than 1 year (*n* = 343, 72.4%).

**TABLE 1:** Socio-demographic and clinical characteristics of participants with paired EDTA and PSC samples attending routine HIV care in selected clinics in Ekurhuleni and Bojanala districts participating in the assessment of the performance of the PSC (2019), South Africa

Participant characteristics	<18 years		≥18 years		Total	
	*N*	%	*N*	%	*N*	%
Gender						
Male	11	55.0	169	35.1	180	35.8
Female	9	45.0	313	64.9	322	64.2
Language						
Setswana	8	40.0	121	25.1	129	25.7
Sesotho	1	5.0	46	9.5	47	9.4
isiZulu	10	50.0	154	32.0	164	32.6
English	1	5.0	144	29.9	145	28.8
isiXhosa	–	–	17	3.5	17	3.4
ART Duration						
<1 year	7	35.0	126	27.6	133	28.0
≥1 year	13	65.0	330	72.4	343	72.0
Virologic Failure, (≥1,000 cp/mL)						
PSC plasma	6	15.8	32	84.2	38	100.0
EDTA plasma	6	13.6	38	86.4	44	100.0

Sample characteristics

### Dried plasma samples

Of the 538 dried plasma samples sent to the laboratory, three were not processed due to insufficient spotting (*n* = 2) and a missing sample in the pack (*n* = 1). From the 535 processed samples, 42 were classified as failures due to sample clot errors, and 32 yielded invalid results due to run control failures (*n* = 27) and invalid quantitation standards (*n* = 5). From these, repeat tests were run for 23 samples. The overall failure rate (pre- and post-analytical rate) for PSC samples was 3.5% (95% CI: 2.1%–5.5%).

### EDTA-plasma samples

Of the 538 EDTA samples sent to the laboratory, 18 were not processed due to incorrect barcodes pasted on the laboratory sheets (*n* = 2), incorrectly requested by HCW (*n* = 14), and blank barcodes (*n* = 2). From the 520 processed, 14 were rejected due to: clinical error (*n* = 2), sample insufficiency (*n* = 1), invalid results (*n* = 6), and unsuitable specimen (*n* = 5). The overall failure rate for EDTA samples was 2.7% (95% CI: 1.5%–4.5%).

### Validity of paired dried plasma samples

A total of 502 paired samples were collected from 10 clinics for VL assessment using PSC and EDTA plasma. Of these, 446 (89%; 95% CI: 86.0%–91.7%) and 502 (99.8%; 98.9%–100.0%) valid test results were obtained from PSC and EDTA plasma, respectively (Table 2). The result from the paired *t* test indicates that there was no significant difference between the means of the paired detectable numerical values between PSC and EDTA with a *t* value of −0.011 and *P*-value of 0.9917. Also, there was no significant difference in the marginal frequencies of the viral suppression binary outcome between both test with a marginal difference in VL proportion of 0.0044 (−0.01 to 0.02) and 0.005 (−0.013 to 0.025) and a *P*-value of 0.48 both in the general and >18 years and older population. The overall mean log_10_ difference in titer between PSC and EDTA plasma was 0.01 log_10_ cp/mL with 95% CI (−0.12 to 0.14)

**TABLE 2:** Frequency of paired samples obtained, valid VL tests conducted, and reported virologic failures in the assessment of the performance of the PSC for HIV VL monitoring in South Africa, 2019

Clinic ID	Paired PSC and EDTA plasma VL samples requested	PSC samples			EDTA-plasma samples		
		Valid results	Virologic failures (≥1,000 cp/mL) N (%)	95% CI	Valid results	Virologic failures (≥1,000 cp/mL) N (%)	95% CI
Tembisa	62	48	2 (4.17)	0.5–14.3	62	4 (6.5)	1.8–15.7
Bapong	66	60	3 (5.0)	1.0–13.9	66	4 (6.1)	1.7–14.8
Ikhutseng	25	16	2 (12.5)	1.6–38.3	25	2 (8.0)	1.0–26.0
Bafokeng	47	45	7 (15.6)	6.5–29.5	47	9 (19.1)	9.1–33.3
Boitekong	16	12	0 (0.0)	0.0–26.5	16	0 (0.0)	0.0–20.6
Vosloorus	74	58	4 (6.9)	1.9–16.7	74	5 (6.8)	2.2–15.1
Katlehong	59	59	6 (10.2)	3.8–20.8	59	6 (10.2)	3.8–20.8
Goba	45	45	3 (6.7)	1.4–18.3	45	2 (4.4)	0.5–15.1
Germiston	60	56	5 (8.9)	3.0–19.6	60	5 (8.3)	2.8–18.4
Daveyton	48	47	6 (12.8)	4.8–25.7	48	7 (14.6)	6.1–27.8
Total	502	446	38 (8.5)	6.1–11.5	502	44 (8.8)	6.4–11.6

### Agreement/disagreement between VLs collected and assessed using PSC and EDTA at 1,000 cp/mL

Overall agreement of VL suppression (<1,000 cp/mL) was observed in 98.2% (*n* = 35 + 403/446) of the samples correctly classified between test results from PSC and routinely collected EDTA plasma (Table 3). Virologic failure (≥1,000 cp/mL) was correctly classified in 35 samples (Table 3). Overall, discordance was observed in eight (1.8%) samples from adult patients (four males and four females), where the sample results were misclassified as virologically suppressed by PSC but as virologic failures by EDTA plasma. One-eighth (12.5%) of the VL measurement was discrepant within 0.5 log_10_ cp/mL. The mean VL for this misclassification was 1,337 cp/mL.

**TABLE 3:** Performance of the PSC for HIV VL monitoring in samples collected from routine patients participating in the study assessing the performance of a PSC for HIV VL monitoring in 2019, South Africa

		EDTA-plasma VL (Gold standard)					
		≥1,000 cp/mL		<1,000 cp/mL		Total	
PSC VL	≥1,000 cp/mL	35		3		38	
	<1,000 cp/mL	5		403		408	
Total		40		406		446	
Sensitivity		87.5% (95% CI: 73.2%–95.8%)					
Specificity		99.3% (95% CI: 97.9%–99.8%)					
Overall agreement		98.2% (95% CI: 96.5%–99.2%)					

### Sensitivity and specificity of PSC in field conditions

Analysis of 446-paired samples at 1,000 cp/mL threshold yielded an overall specificity of 99.3% (95% CI: 97.9%–99.8%) and sensitivity of 87.5% (95% CI: 73.2%–95.8%; Table 3). Specificity and sensitivity in detecting virologic failure using PSC in children were 100%, and in adults, specificity was 99.2% (95% CI: 97.8%–99.8%), and sensitivity was 87.5% (95% CI: 68.9%–95.0%).

### Usability rating of PSC in blood collection for VL monitoring by healthcare and laboratory staff

**Fig 1:**
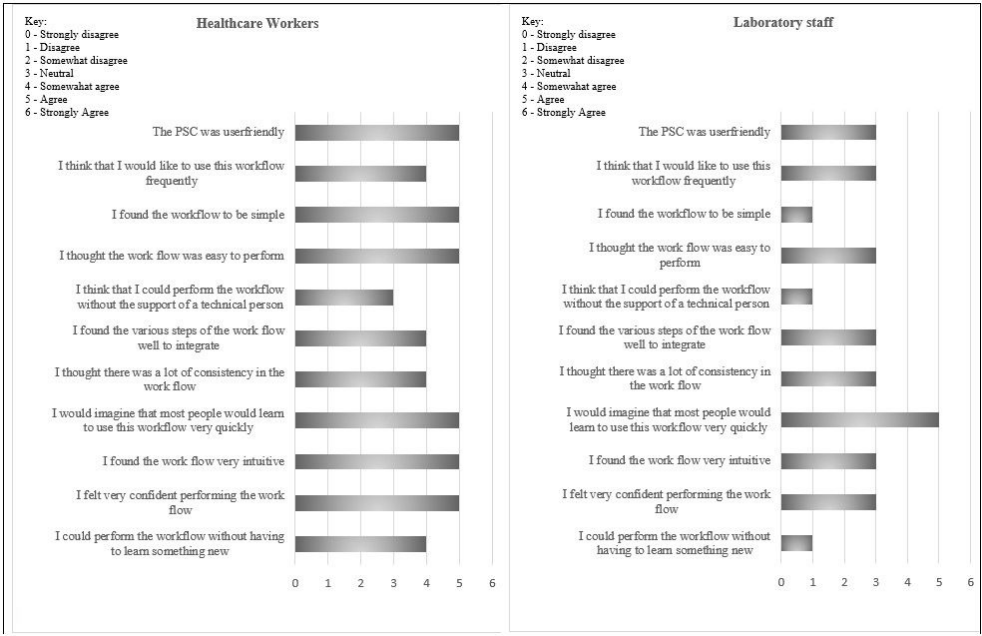
User rating of different workflow sub-tasks among HCWs (*n* = 8) evaluating the collection and spotting of the PSC (Fig. 1a) and laboratory staff (LAB; *n* = 5) assessing the processing of the PSC for VL (Fig. 1b) participating in the study assessing the performance of a PSC for HIV VL monitoring in 2019, South Africa, (numbers represent a mean score).

HCWs staff scored most of the sub-tasks below 4 (somewhat agree) but most agreed that people would learn to use PSC workflow quickly (above 4; Fig. 1a). Initially, these staff had some difficulties obtaining sufficient samples, determining when the capillary was adequately filled with blood, collecting blood samples without the introduction of air bubbles in the capillary tubes, and spotting the correct amount of blood onto the wells in the PSC. Laboratory staff did not think that PSC could be used without the support of a technical person, scoring this sub-task 1 (“disagree”; Fig. 1b).

## DISCUSSION

Findings from our field evaluation showed that the PSC was suitable for the monitoring of HIV VL as the mean difference of log-transformed VL PSC-plasma results and the matched EDTA-plasma samples were −0.01 with 95% CI (−0.15 and 0.12). The study results indicate acceptable sensitivity (87%), high specificity (99%), and an overall high agreement (98.2%) for detecting HIV VL in PSC-plasma samples of PLHIV engaged in care. This is an important finding especially in settings with high HIV burden and limited access to VL tests where using PSCs may improve HIV VL monitoring. Our findings from the 446 paired samples in clinic settings were comparable to previous findings of 485 paired samples in a laboratory setting (7).

PSC- and EDTA-plasma VLs were comparable, with a concordance of 446 (98.2%) virologic failure in samples correctly classified. Differences in VL quantification thresholds between PSC and EDTA plasma as claimed by the manufacturer were evident, as EDTA-plasma reported VL copies as low as 20 cp/mL, while PSC-plasma reported quantified VL only at 730 cp/mL and above in this study (7). This relatively high lower LoD may limit the use of PSC in South Africa where clinical guidelines recommending patient management are based on VL counts of <50 cp/mL. As with virologic failure at the cut-off point of 1,000 cp/mL, PSC showed to be very specific. A high specificity overall implied reliability for the correct classification of VL suppression. These findings were comparable to those of similar studies conducted in Mozambique (12), Kenya (13), and Uganda (14). Furthermore, agreement between these sample types was similar for both adults and children, indicating that PSC and EDTA plasma were equally comparable. A high concordance was observed in the studies conducted among PLHIV in Mozambique, Uganda, and Kenya and a study using stored blood specimen in South Africa by Hans et al. (15). The diagnostic performance of the PSC card in children was good with a sensitivity of 100%. However, given the few number of children enrolled in the study, these results should be interpreted with caution, and further research with sufficient samples of children (<18 years) needs to be conducted to verify.

Our findings highlighted the total VL misclassification rate of 0.7% where three samples were classified as virologic failures and five as virally suppressed by the PSC. The misclassification rate was similar to findings from Mozambique by Vubil et al. (12). This finding was considerably lower than that found in many reports for DBS analyses (10%–15%) including a recent study evaluating the PSC in laboratory settings (3%) (7). Eight (14) samples were misclassified between PSC and EDTA plasma, which is different from Carmona et al. (7) study findings where 14 specimens with discordant EDTA and PSC results had a VL of around 1,000 cp/mL, with a median log10 titer of 3.13 (7). The lower misclassification rate could be due to the performance of the Cobas PSC which does not support replication of RNA (7, 12). This finding confirms the suitability of the PSC as an alternative VL diagnostic test in areas where preserving stored plasma may be challenging (14).

Similar to a previous study, usability findings revealed acceptability and preferences by nurses and laboratory staff to use PSC for VL monitoring for all patients (7, 16). Phlebotomists and nurses rated the sample collection favorable and indicated the usefulness of the PSC in their setting. Additionally, the sub-tasks in the pre-analytical workflow were rated neutrally by laboratory staff. Overall, the sample collection and the pre-analytic workflow of the PSC appeared to be of low complexity and user-friendly, especially once staff became familiar with the procedures. Multiple spots on the card allow for repeat testing of spots in the event of failed/invalid samples. The usability findings, however, highlighted areas that required further training on the use of the card. The correct use of capillary tubes to spot the cards is an area of concern to avoid insufficient blood samples, blood clots, or perforation of the membrane. The rates of rejection due to invalid samples caused by clotted blood samples are likely to be reduced when HCWs follow correct blood collection procedures. Since most HCWs are familiar with collecting DBS samples, this skill may be transferred to PSC use. Further retraining is needed to augment these skills. Acceptability of PSC was high, and most workflows were executed once the phlebotomists became familiar with the use of capillary tubes, and their performance improved markedly.

The main strength of this study was the clinical evaluation of the PSC card for VL monitoring and addition to the growing literature on the field evaluation of the PSC in studies in sub-Saharan Africa. Similar studies have been conducted in the laboratory using samples collected from routine settings, and concordance between the PSC- and EDTA-plasma samples was found (12–14). A cross-sectional study in Uganda found HIV-1 drug resistance genotyping success rate for DBS and plasma of 49.8% and 85.9%, respectively (17). The latter is closer to findings in our study of 89% and 99.8% valid test results for PSC and EDTA-plasma, respectively, and furthermore, PSCs were deemed suitable for HIV-1 genotypic drug resistance testing in South Africa (18). Our study was not without limitations; first, we were unable to reach the desired sample size due to difficulties in accessing some clinics for various reasons. However, the sample size reached was comparable to the sample size of the previous study conducted in the laboratory (7) which showed similar concordance between EDTA- and PSC-plasma samples. Second, we were unable to reach the preferred sample size proposed for children. As such, interpreting the specificity and sensitivity values should be done with caution, especially in relation to children. A similar study in the future may consider targeting data collection during school holidays. Third, 18% of PSCs were processed beyond 2 months after collection. Although we were unable to accurately calculate the turn-around time, the stability of the card was confirmed as 82% of the samples were tested within 56 days, and a low misclassification rate was reported. Despite these limitations, this study provides an opportunity to conduct further research that includes a large sample size to assess the cost associated with VL monitoring, evaluation of sub-tasks among HCWs in urban and rural settings, and the possibility of using PSC for other blood-borne pathogen nucleic quantification.

### Conclusions

Findings from this study suggest that PSC- and EDTA-plasma VLs were comparable, with both concordance in the classification of virologic failure and the quantitative agreement. Differences in test thresholds for VL measures between PSC and EDTA plasma were evident, as EDTA plasma had the capacity to measure VL copies of as low as 20 cp/mL, while PSC had the capacity to only measure at 738 cp/mL in this study. Furthermore, agreement between these tests was similar for both adults and children, indicating that PSC and EDTA plasma were equally comparable. This implies that PSC is a reliable alternative for VL monitoring especially in areas where it may be logistically challenging to rely on EDTA plasma.
